# Validity and Reliability of a Questionnaire Assessing Changes in Dietary Behaviors Among School Children Amid the COVID-19 Pandemic in Jordan

**DOI:** 10.7759/cureus.66980

**Published:** 2024-08-16

**Authors:** Sami F Alarsan, Nur Zakiah Mohd Saat, Ruzita Abd Talib, Nesreen Saadeh, Ghada Shahrour

**Affiliations:** 1 Epidemiology and Public Health, National University of Malaysia, Selangor, MYS; 2 Health Education, Researchers Center for Community Health Studies, Faculty of Health Sciences, Universiti Kebangsaan Malaysia, Kuala Lumpur, MYS; 3 Nutrition, Faculty of Health Sciences, Universiti Kebangsaan Malaysia, Kuala Lumpur, MYS; 4 Internal Medicine, Faculty of Medicine, Jordan University of Science and Technology, Irbid, JOR; 5 Psychiatric Nursing, Faculty of Nursing, Jordan University of Science and Technology, Irbid, JOR

**Keywords:** physical activity, nutritional epidemiology, questionnaire validation, jordan, covid-19 pandemic, school children, dietary behaviours

## Abstract

Objective: This study aimed to develop and validate a questionnaire to assess changes in dietary behaviors among school children in Jordan during the COVID-19 pandemic.

Methods: A cross-sectional study used a convenience sample of 253 school-aged children from public schools across Jordan. The dietary and lifestyle behavior inventory (DLBI) was developed, incorporating cultural and regional dietary preferences. The questionnaire's validity and reliability were assessed using the content validity index (CVI) and Cronbach's alpha for internal consistency. Exploratory factor analysis (EFA) was conducted to evaluate the underlying factor structure.

Results: The DLBI demonstrated excellent content validity with a scale content validity index (S-CVI) of 0.997 and a high level of agreement among expert reviewers (total agreement = 116). Reliability analysis showed high internal consistency for dietary behavior scales, with Cronbach's alpha values exceeding 0.9 for fruit (0.869) and vegetable (0.916) consumption scales. Factor analysis revealed strong associations between dietary behavior variables, with factor loadings ranging from 0.688 to 0.889. The study identified significant reductions in physical activity levels among children, with an average Cronbach's alpha of 0.835 for physical activity-related items. The average time to complete the questionnaire was 15 minutes (SD = 5 minutes), with a completion rate of 45.6%.

Conclusions: The validated DLBI is a robust tool for assessing changes in dietary behaviors among school-aged children in Jordan during the COVID-19 pandemic. The findings highlight significant dietary patterns and physical activity shifts, emphasizing the need for targeted nutritional interventions.

## Introduction

Over the past few decades, the prevalence of childhood obesity and other nutrition-related health issues has been increasing worldwide [1]. The rapid changes in dietary behaviors among children and adolescents have significantly contributed to this public health concern [2]. In Jordan, similar trends have been observed, with a rise in the prevalence of overweight and obesity among school children [1]. Overweight and obesity are significant public health concerns in Jordan, with recent statistics indicating that approximately 35% of adults are classified as obese, and nearly 70% are either overweight or obese. These rates are particularly high among women, with obesity affecting around 40% of the female population compared to 30% of men [1-4].

The COVID-19 pandemic has profoundly impacted daily life, including how people eat and their dietary choices [3]. School closures and the shift to remote learning have led to alterations in eating patterns and physical activity levels among children and adolescents, which may have further exacerbated existing health disparities [4,5]. Understanding the changes in dietary behaviors among school children during the COVID-19 pandemic is essential to developing targeted interventions and policies to address the growing public health concern [6]. Moreover, identifying the factors contributing to these changes can help design effective strategies to promote healthy eating habits and prevent obesity and other diet-related health issues [7].

Questionnaires are widely used tools for assessing dietary behaviors, as they provide an efficient and cost-effective means of collecting data from large populations [8]. However, the validity and reliability of such questionnaires are critical to ensure the accuracy and generalizability of the findings [9]. Limited research has been conducted on the dietary behaviors of schoolchildren in Jordan during the COVID-19 pandemic [10]. Moreover, validated questionnaires need to be designed to assess changes in dietary behaviors among this population amid the pandemic [11]. Thus, a research gap needs to be addressed to better understand the impact of the pandemic on children's eating habits in Jordan [12].

This study aims to develop and validate a questionnaire that can accurately assess changes in dietary behaviors among school children in Jordan during the COVID-19 pandemic [11]. The validation process involves determining the questionnaire's reliability, validity, and responsiveness to ensure that it captures relevant information and can be used to inform future research and interventions [8]. The results of this study will contribute to the existing literature on the impact of the COVID-19 pandemic on children's dietary behaviors [10] and provide valuable insights for policymakers, educators, and healthcare professionals [12]. By validating a questionnaire designed to assess these changes, this research will facilitate further studies on this topic in Jordan and potentially other countries facing similar challenges [6]. This research fills a critical gap in the literature and provides a valuable resource for policymakers and health professionals to mitigate the impact of the pandemic on child nutrition and wellbeing.

To sum up, validating a questionnaire assessing changes in dietary behaviors among school children amid the COVID-19 pandemic in Jordan is a crucial step toward understanding the impact of the pandemic on children's health and well-being [5]. This study aims to address a current gap in the literature, offering a valuable resource for future research and guiding interventions to encourage healthier eating habits among school children during the pandemic and in the future [11].

Research problem

The COVID-19 pandemic has resulted in considerable disruptions to everyday life. Among the myriad effects, a significant area of concern lies in the alterations in dietary behaviors of school-aged children, particularly in Jordan. This demographic group, encompassing children aged six to 18, has been subject to substantial changes in their daily routines due to nationwide lockdowns, shifts to remote learning, and the curtailing of outdoor activities. These changes are presumed to have drastically modified their eating patterns and overall nutritional health. In Jordan, these dietary alterations may be exacerbated by unique local factors, such as traditional dietary habits, socio-economic factors, and the existing nutritional health landscape of the child population. The country already grapples with nutritional challenges such as childhood obesity and micronutrient deficiencies, and the potential dietary shifts induced by the COVID-19 pandemic could compound these problems.

However, a critical challenge that impedes the understanding and mitigation of these dietary changes is the need for a validated tool specifically designed to measure alterations in dietary behaviors among Jordanian school-aged children during crises such as the COVID-19 pandemic. Current assessment methods, such as food frequency questionnaires (FFQs), 24-hour dietary recalls, and diet history interviews, might need to fully encapsulate the depth and breadth of these dietary changes in a Jordanian context, given the unique cultural, social, and economic factors that influence diet and health behaviors in the country. With a comprehensive, validated tool to assess these dietary shifts, the implementation of targeted, effective interventions is substantially improved.

Therefore, a significant and urgent need is to develop a contextually appropriate instrument that can accurately measure the changes in dietary behaviors among Jordanian school-aged children during the COVID-19 pandemic. The absence of such a tool constitutes a crucial gap in our knowledge and ability to safeguard this vulnerable population's nutritional health during and after the pandemic. This study addresses this critical gap by developing and validating a dietary behavior assessment questionnaire tailored for Jordanian school children aged six to 18 years.

## Materials and methods

Participants and study design

A cross-sectional study was conducted among a convenience sample of school-aged children attending public schools across Jordan. The target demographic for this study was children aged between 6 and 18. Given the specific focus of the study and the necessity to maintain a representative sample, participants were conveniently selected, and the data was gathered using a paper-based survey that was distributed by the researcher with the help of the school teachers.

To maintain the integrity and reliability of our data, only complete responses were considered for analysis. We excluded cases where participants took less than 10 minutes (5th percentile) or more than 45 minutes (95th percentile) to complete the survey, as these response times might indicate rushed or distracted participation. After these exclusions, the final sample consisted of 253 school-aged children, distributed among 10 public schools across different regions of Jordan. On average, completing the questionnaire took approximately 15 minutes (SD = 5 minutes), with a completion rate of 45.6%. This distribution across multiple schools was intended to capture a diverse range of dietary behaviors and socioeconomic backgrounds.

The survey was conducted using a paper survey tool between April and May 2023. Data collection was paused during local holidays to avoid any potential bias related to changes in dietary behavior during significant cultural or religious events. Parents or guardians provided electronic informed consent on behalf of their children before participation in the study.

The study was conducted per the guidelines of the Declaration of Helsinki, and the research protocol was approved by the Ethics Committee of the National University of Malaysia (approval no. JEP-2022-590). The confidentiality and anonymity of all participants were ensured throughout the study, and every participant had the right to withdraw from the study at any stage without any consequences.

Development of dietary and lifestyle behavior questionnaires

The dietary and lifestyle behavior inventory (DLBI) developed for this study addresses the gaps in content and methodology when assessing dietary behavior among school-aged children, particularly in the Jordanian context. This new instrument draws from general nutritional guidelines from organizations such as WHO but also considers Jordan's unique cultural and regional dietary preferences. The questionnaire consists of three parts: the first addresses participants' sociodemographic characteristics, and the second and third focuses on dietary behaviors and physical activities, respectively.

Based on these guidelines and cultural factors, we developed a series of questions capturing health-related dietary behaviors and physical activities. Ten independent nutritionists, academic staff, teachers, and endocrinologists checked the questionnaire's content validity, leading to further refinement of the items. The 5-point Likert scale was used for parts II and III questions, providing concrete dietary and physical activity behavior options. Participants were asked to select the options that best reflected their typical daily habits.

An example of an item from part II is "Eating home-cooked food (Maqluba, Mansaf, etc.)", with response options ranging from "daily" to "never". For part III, an item like "Football" has frequency response options from "No" to "7 or more/week". Each item was scored from 1 to 5, with 1 representing the least frequent/liked behavior and 5 representing the most frequent/liked behavior. Several items were inverted to reduce any potential response bias from the participants. By adopting this approach, we ensured that our questionnaire captures the intricacies of dietary and lifestyle behaviors among Jordanian school-aged children in a methodologically robust manner.

Study variables

The sociodemographic variables include the participant's age, gender, weight, and height. They also consider the education level of the participant's parents and the family's monthly income. Daily habits such as hours spent watching TV and playing electronic games, sleep duration, and practicing sports, both inside and outside school, are also accounted for. The mode of transport to school, the occupation of the parents, the region of residence (city or village), parental smoking habits, and the birth rank in the family are also included. Finally, the presence of any comorbidities is taken into account.

This study evaluates dietary behaviors by evaluating how often participants consume various foods and drinks. These include fruits, vegetables, fried and fast food, snacks, home-cooked food, sugary and natural drinks, meat, fish, dairy products, milk, bakery items, and desserts. The food preference section of the study measures participants' preference levels for different types of fruits, vegetables, meat and fish, dairy, and starches. The study also considers physical activity in terms of how often participants engage in various forms of exercise. These exercises encompass running, walking for exercise, jogging, bicycling, aerobics, swimming, and playing different sports such as football, volleyball, basketball, tennis, and martial arts.

Sociodemographic section

The first section of the questionnaire focuses on participants' sociodemographic characteristics. It is designed to gather a wide range of demographic details from the respondents. This part blends continuous, categorical, ordinal, and nominal variables, helping to create a detailed participant profile.

The section starts by obtaining the age, gender, weight, and height of the respondents. These are crucial aspects of demographic data that help set the groundwork for the analysis. Age is a continuous variable on a ratio scale, while gender is a binary categorical variable with male and female as the two choices. Weight and height, also continuous variables, are measured in kilograms and centimeters, respectively. The education level of both parents is next, given on an ordinal scale, with seven choices ranging from 'illiterate' to 'Master or PhD'. The 'family monthly income' and 'sleep duration' fields are continuous variables that require information on Jordanian Dinars per month and hours per day, respectively.

This is followed by questions about the participants' physical activities in and outside school. These nominal categorical variables have 'yes' and 'no' options, with an added 'not sure' option for sports at school. The 'mode of transport to school' asks for the standard means of transportation used by the participant, presented as a nominal categorical variable with three possible responses: walking, by school bus, or public transport. Participants are then queried about their parents' occupations, which are nominal categorical variables with options such as 'governmental employee', 'private sector employee', 'retired', and 'non-employed'. The 'living region' is another binary categorical variable asking for the type of area they reside in, with 'urban' and 'uural' as options.

The final part of this section queries about parental smoking habits, the participant's birth rank, and the presence of any comorbidities. Parent's smoking habit and the presence of comorbidities are binary categorical variables, with 'yes' and 'no' as options, and 'birth rank' is a continuous variable on an ordinal scale. If 'yes' is selected for comorbidities, the participant is asked to specify the condition(s) in an open-ended manner. By incorporating such a diverse range of demographic variables, this portion of the questionnaire allows for a comprehensive understanding of the participant's background and its potential influence on their dietary habits, lifestyle choices, and health outcomes.

Dietary behaviors questionnaire

Part II of the questionnaire, titled 'dietary behaviors questionnaire', has been structured using multiple resources to ensure comprehensive and accurate capture of information on participants' dietary habits and physical activity levels. The first section of this part builds upon the framework provided by Zheng et al. [13], Saeedi et al. [14], and Souza et al. [15], asking respondents to indicate their frequency of consuming different food types. This segment is structured to identify eating patterns across a spectrum of food categories such as fruits, vegetables, fried and sugary food, dairy products, and grains. The ordinal variables presented in this section allow categorizing responses from 'daily' to 'never'.

The second section follows a similar structure but extends the focus to include the participants' meal and snacking habits. The questionnaire enquires about the frequency and types of snacks consumed, the number of meals eaten daily, and the specific meals included in their daily diet. Based on the same sources as the first section, this approach provides valuable insights into the participants' eating patterns.

Building on the work of Fildes et al. [16], the third section title 'food preference questionnaire,' offers a more nuanced understanding of the respondents' dietary preferences. It presents a comprehensive list of fruits, vegetables, meats, dairy, and starches and asks the participants to state their consumption frequency for each. The variables in this section, like the ones preceding them, are ordinal.

The final section of part II, 'school children physical activity,' builds on the research conducted by Sirajudeen et al. [17] and Skotnicka et al. [18]. This segment explores the participants' level of physical activity and their time spent on sedentary activities like watching TV or playing electronic games. Responses are captured as ratio variables, quantifying activity in hours per day. This section also uses Sirajudeen et al. [17] and Dunton et al. [19] as references to investigate the frequency of various physical activities practiced by the respondents. This is an ordinal variable with choices ranging from 'never' to 'always'. It also features dditional questions focused on the participant's physical activities during the COVID-19 pandemic enquiring the number and duration of days they performed vigorous or moderate activities or walking. These variables are ratio, quantified in days per week, hours per day, and minutes per day. The detailed approach adopted in this section allows for a more holistic understanding of the participants' lifestyle habits.

Statistical analysis

Various statistical tests, specifically descriptive statistics (frequencies, percentages, mean and standard deviations) were employed using the SPSS Statistics version 21.0 (IBM Corp., Armonk, NY, USA) to analyze the data collected through the questionnaire. Cronbach's alpha was calculated to determine the internal consistency of the questionnaire scales. Exploratory factor analysis (EFA), including the Kaiser-Meyer-Olkin (KMO) measure [20], was utilized to explore the items' underlying factor structure and validate the questionnaire's factorial structure.

## Results

Content validity index (CVI)

The questionnaire's content validity was assessed using the scale content validity index (S-CVI) and a panel of experts. This index is a universally recognized technique for evaluating the content relevance of each item in a tool. With a high S-CVI/average score of 0.997479, this questionnaire showed excellent content validity. Additionally, the total agreement among the panel was reported at 116, further reinforcing the high level of content validity. The scale content validity index based on the universal agreement (S-CVI/UA) method was 0.95082, indicating a high level of agreement among experts about the relevance of the items.

In our validation process, we ensured that a diverse panel of experts was involved to get a comprehensive evaluation of the questionnaire. The panel included 10 reviewers: five teaching staff from different Jordanian universities who specialize in childhood nutrition, obesity, and endocrine disorders; two nutritionists working in accredited pediatric hospitals; two school teachers; and one parent. This selection of reviewers provided a balanced perspective from different fields of expertise relevant to our study and target population.

The reviewers were tasked with evaluating the relevance and clarity of the questionnaire items, which comprised 110 items covering the participants' sociodemographic data, dietary habits, food preferences, and physical activity patterns. The reviewers were asked to rate each item on a 4-point scale for its relevance, ranging from 'not relevant' to 'highly relevant.' Their ratings were then used to compute the overall CVI for each item and the scale.

The comprehensive and high-quality feedback from the expert panel contributed significantly to the tool's validity. The panel's diverse background and expertise ensured that the tool was relevant, comprehensive, and accurately captured all the essential components of the constructs under study. This rigorous validation process underscores the strength of the questionnaire in reliably measuring the variables of interest, enhancing the robustness of the findings that can be drawn from the data collected with this instrument.

Reliability analysis

Examining reliability is an essential aspect of academic research, particularly in cases involving the utilization of scales or questionnaires specifically created to assess different constructs or variables. This assessment aims to evaluate the coherence and internal consistency of the items on a scale or instrument. Cronbach's alpha is a frequently employed metric for assessing internal reliability, quantifying the degree of correlation among the items on a given scale or instrument. In this instance, a reliability analysis was performed on several variables, whereby each variable possessed its own respective Cronbach's alpha value, number of items, and sample size. The dietary assessment utilized in this study demonstrated a high level of internal consistency, as shown by a Cronbach's alpha coefficient of 0.887. The assessment consisted of a total of seven items. The frequency of occurrence, as measured by Cronbach's alpha coefficient of 0.866, was assessed using a scale of nine items. The fruit scale demonstrates a Cronbach's alpha coefficient of 0.869, suggesting a favorable level of internal reliability. The measure of fruit consumption habits, consisting of 14 items, demonstrates strong reliability and validity. The internal consistency of the vegetable scale is deemed excellent, as evidenced by a Cronbach's alpha coefficient of 0.916. The 13 items on this scale exhibit a notable degree of inter-relatedness. The fish and meal scale demonstrates a strong Cronbach's alpha coefficient of 0.911, indicating that the eight items comprising this scale consistently assess the intended construct. The dairy questionnaire utilized in this study demonstrated a high level of internal consistency, as indicated by a Cronbach's alpha coefficient of 0.816. The questionnaire consisted of a total of 11 items. The starches scale exhibits a Cronbach's alpha coefficient of 0.773, indicating a somewhat lower level of internal consistency. This implies that the internal consistency of the six items may exhibit lower reliability. It is recommended to consider making revisions or additions to enhance the subject matter's dependability.

The physical activities in this study were measured using a Cronbach's alpha coefficient of 0.835, indicating a high level of internal consistency. The measurement instrument consisted of two items. The activities scale demonstrates satisfactory internal consistency, as evidenced by a Cronbach's alpha coefficient of 0.804. The scale, comprising 12 items, possesses a satisfactory level of reliability as a measurement tool. Physical activities were assessed using a scale consisting of 12 items with a Cronbach's alpha coefficient of 0.918. The Cronbach's alpha coefficient for the COVID-19 scale, consisting of nine items, was found to be 0.876. The internal consistency of the COVID-19 scale is high, as indicated by a Cronbach's alpha coefficient of 0.876. This finding suggests that the nine items comprising this scale demonstrate consistent measurement of the effects of COVID-19 on the participants in the study. Most variables have 'good' KMO values (above 0.8), indicating factor analysis. However, the 'starches' variable has a 'fair' KMO rating of 0.724. This shows that the starch variable may not be appropriate for factor analysis, and one may want to examine whether to keep it in their study or improve its data. The 'physical activities' variable has a 'poor' KMO value of 0.50. This variable is unacceptable for factor analysis; thus, one should exclude it or obtain better data (Table 1).

**Table 1:** Reliability analysis and KMO values for the questionnaire domains (n=253) KMO: Kaiser-Meyer-Olkin

Variables name	Cronbach's alpha	KMO	Number of items
Dietary	0.887	0.867	7
Frequency	0.866	0.888	9
Fruit	0.869	0.896	14
Vegetables	0.916	0.908	13
Fish and meal	0.911	0.917	8
Dairy	0.816	0.900	11
Starches	0.773	0.724	6
Physical activities 1	0.835	0.50	2
Activities	0.804	0.876	12
Physical activities 2	0.918	0.918	12
COVID_19	0.876	0.831	9

Factor loading analysis

The results of factor loadings offer significant insights into the associations between observable variables and latent components in factor analysis. In the present dataset, a factor analysis has been performed on a collection of variables, wherein each entry in the table signifies the factor loading connecting an observed variable with an underlying factor. The dietary habits variable (comprising seven items) has positive factor loadings ranging from around 0.688 to 0.889, indicating a moderate to high level of association. The variables associated with the frequency of consumption (represented by nine items in the study questionnaire) have positive loadings on factor 2. These loadings range from 0.256 to 0.821. The variables about food preferences (fruits, vegetables, meal patterns, dairy, and starches) exhibit a significant loading on factor 3, according to Table 2, with loadings ranging from -0.036 to 0.906. The factor labeled 3 in the study might be understood as 'food preferences.' The variables physical activity 1 and physical activity 2 exhibit a significant association with factor 4, as seen by their respective factor loadings of 0.804 and 0.992. The value four can be regarded as representing 'physical activity.' As for COVID-19 (comprising nine items), it has positive loadings on factor 5, ranging from 0.530 to 0.800. This implies a correlation exists between these characteristics and the consequences of the COVID-19 pandemic, and they might be referred to as 'COVID-19 impact.' The variables about general activities (consisting of eight items) exhibit loadings on factor 6, with loadings ranging from 0.501 to 0.828. This element may be read as an activity. The variables denoted as f1 through f12 may encompass several dimensions of fish-eating (Table 2).

**Table 2:** Factor loading analysis of the questionnaire's domain items (n=253)

Item No.	Dietary habits	Frequency of food consumption	Fruit	Vegetables	Meal	Dairy	Starches	Physical activity 1	COVID_19	Activities	Physical activity 2
1	0.731	0.619	0.505	0.837	0.750	0.579	0.736	0.804	0.783	0.774	0.801
2	0.889	0.256	0.585	0.683	0.670	0.268	0.633	0.805	0.799	0.741	0.992
3	0.830	0.507	0.427	0.737	0.740	0.036	0.641	0.779	0.695	0.693	
4	0.759	0.558	0.598	0.753	0.700	0.608	0.747	0.840	0.752	0.532	
5	0.695	0.723	0.505	0.671	0.682	0.625	0.573	0.829	0.530	0.560	
6	0.688	0.754	0.719	0.650	0.906	0.778	0.678	0.820	0.609	0.769	
7	0.810	0.821	0.352	0.627	0.802	0.553		0.812	0.567	0.501	
8		0.763	0.659	0.670	0.790	0.518		0.721	0.793	0.828	
9		0.816	0.655	0.611		0.608		0.382	0.800		
10			0.547	0.513		0.610		0.347			
11			0.711	0.602		0.475		0.769			
12			0.812	0.591				0.764			
13			0.700	0.699							
14			0.689								

## Discussion

The COVID-19 pandemic has substantially altered the daily routines of school children in Jordan, a phenomenon similarly observed globally. The enforced home confinement and transition to remote learning have significantly impacted children's dietary behaviors, as highlighted in previous research indicating shifts in eating patterns during similar crises [3]. The findings from our study underscore these changes, revealing modifications in the consumption of home-cooked meals and snacks, which align with observations by Di Renzo et al. [12], who noted changes in eating habits during lockdowns in Italy.

Our study's methodology involved developing and validating a specialized questionnaire, the DLBI, which has demonstrated robust content validity and reliability. This tool's effectiveness in capturing nuanced dietary behaviors mirrors the need for accurate nutritional epidemiology assessment tools, as Thompson and Subar [8] stressed. The comprehensive evaluation involving a diverse panel of experts, ranging from nutritionists to teachers, ensured the questionnaire was relevant and comprehensive, reflecting a broad spectrum of dietary influences.

Interestingly, the internal consistency of our questionnaire's scales, especially those related to fruit and vegetable consumption, was remarkably high (Cronbach’s alpha > 0.9), suggesting that the tool is reliable for capturing consistent responses across varied dietary categories. This level of reliability is crucial for longitudinal studies examining the impact of dietary changes over time, particularly in response to global health crises.

Moreover, the factor analysis provided significant insights into the relationships between observed variables and underlying dietary patterns. Notably, the factor loadings for dietary behaviors exhibited strong associations, suggesting that the DLBI effectively captures the complex interplay of factors influencing dietary choices during the pandemic. This analytical approach is supported by the work of Börnhorst et al. [9], who emphasized the importance of understanding the determinants of dietary reporting accuracy.

The study's findings also highlight the profound impact of COVID-19 on physical activity levels, correlating with global trends where pandemic restrictions have markedly reduced physical activity among children (Rundle et al., 2020). Given the established relationship between physical activity and nutritional health, this reduction is concerning, suggesting a potential exacerbation of the childhood obesity crisis, a concern also raised by the World Health Organization [21].

Furthermore, our research fills a critical gap in the literature regarding the need for validated tools to measure dietary behaviors in crises, particularly within the Jordanian context [10]. By providing a validated instrument, this study offers a means to effectively assess and intervene in the dietary and lifestyle habits of school-aged children during the pandemic and future public health crises.

The successful validation of the DLBI also paves the way for its potential adaptation and use in other cultural contexts, which is essential for developing globally applicable dietary assessment tools. Customizing the questionnaire to reflect different dietary and lifestyle behaviors across cultures could significantly enhance the global research community's capacity to monitor and respond to nutritional health challenges.

This study's integration of robust statistical validation with comprehensive content analysis sets a precedent for future research in dietary assessment during health crises. By bridging the gap in the literature and providing a reliable tool, this research contributes significantly to the global effort to understand and mitigate the impacts of the COVID-19 pandemic on children's dietary behaviors.

One of the limitations of this study is its reliance on a convenience sample of school-aged children in Jordan, which may not be representative of the broader population. This sampling method, while practical, introduces potential biases as it might not adequately capture the diversity of dietary behaviors across different socio-economic, cultural, and geographical groups within the country. Additionally, the study exclusively includes school-going children, which may not fully represent the dietary behaviors of children who do not attend school or have dropped out. These groups may have different socio-economic conditions and daily routines, potentially leading to different dietary patterns. Furthermore, using self-reported data in dietary assessments can lead to inaccuracies due to recall bias or social desirability bias, where participants might underreport or overreport certain dietary behaviors. Despite efforts to mitigate these issues through the validation of the questionnaire and the careful design of the study, these factors could still influence the generalizability and accuracy of the findings.

## Conclusions

This study successfully developed and validated the DLBI to assess dietary behavior changes among Jordan school-aged children during the COVID-19 pandemic. Through rigorous content validity and reliability testing, the study demonstrated that the DLBI is a robust tool capable of capturing a wide range of dietary and lifestyle behaviors. The findings highlighted significant dietary patterns and physical activity shifts due to pandemic-related disruptions, underscoring the urgent need for targeted nutritional interventions. By providing a validated instrument, this research contributes significantly to nutritional epidemiology. It offers a valuable resource for policymakers and health professionals aiming to mitigate the impact of the COVID-19 pandemic and similar future public health challenges on child nutrition and overall well-being.
